# Association of Preoperative Chemosensitivity With Postoperative Survival in Patients With Resected Gastric Adenocarcinoma

**DOI:** 10.1001/jamanetworkopen.2021.35340

**Published:** 2021-11-19

**Authors:** Lei Deng, Adrienne Groman, Changchuan Jiang, Stuthi Perimbeti, Emmanuel Gabriel, Moshim Kukar, Sarbajit Mukherjee

**Affiliations:** 1 Department of Medicine, Roswell Park Comprehensive Cancer Center, Buffalo, New York; 2 Department of Biostatistics, Roswell Park Comprehensive Cancer Center, Buffalo, New York; 3 Department of Surgery, Mayo Clinic, Jacksonville, Florida; 4 Department of Surgical Oncology, Roswell Park Comprehensive Cancer Center, Buffalo, New York

## Abstract

**Importance:**

Considering its low completion rate, the survival benefit associated with postoperative chemotherapy (PC) is unclear in patients with resectable gastric adenocarcinoma who received preoperative chemotherapy.

**Objective:**

To determine whether preoperative chemosensitivity is associated with postoperative survival among patients with resectable gastric adenocarcinoma who receive PC.

**Design, Setting, and Participants:**

This national, hospital-based cohort study used data from the National Cancer Database, which covers more than 70% newly diagnosed gastric adenocarcinomas in the US, between 2006 and 2017. Participants included patients with clinical stage II or III disease treated with preoperative chemotherapy and curative-intent resection, excluding radiotherapy. Preoperative chemosensitivity was defined as very sensitive (ypT0N0), sensitive (pathological TNM stage less than clinical, excluding ypT0N0), and refractory (pathological greater than or equal to clinical). Data were analyzed in April 2021.

**Exposures:**

Receipt of PC or not.

**Main Outcomes and Measures:**

Overall survival from surgical discharge.

**Results:**

This study included 2382 patients (1599 men [67%]; median [IQR] age, 63 [54-70] years). Most patients (1524 patients [64%]) received no PC. Most patients (1483 patients [62%]) had refractory disease, followed by sensitive disease (727 patients [31%]) and very sensitive disease (172 patients [7%]). Patients with older age (odds ratio [OR], 0.99; 95% CI, 0.97-1.00), comorbidity (OR, 0.71; 95% CI, 0.57-0.90), longer time from chemotherapy initiation to surgery (OR, 0.99; 95% CI, 0.97-1.00), less sensitivity to preoperative chemotherapy (very sensitive vs refractory OR, 0.58; 95% CI, 0.37-0.89; sensitive vs refractory OR, 0.96; 95% CI, 0.76-1.20), and longer surgical hospitalization (OR, 0.95; 95% CI, 0.93-0.97) had a significantly lower likelihood of receiving PC. PC was not associated with improved survival in the whole group (hazard ratio [HR], 0.88; 95% CI, 0.75-1.02). Patients with refractory disease had the worst survival compared with patients with sensitive disease (HR, 0.39; 95% CI, 0.32-0.46) and those with very sensitive disease (HR, 0.12; 95% CI, 0.07-0.20). Preoperative chemosensitivity was significantly associated with the survival benefit from PC (*P* for interaction = .03). PC was significantly associated with longer survival in patients with sensitive disease (5-year survival rate, 73.8% in the PC group vs 65.0% in the no PC group; HR, 0.64; 95% CI, 0.46-0.91), but not in those with very sensitive disease (5-year survival rate, 80.0% in the PC group vs 90.8% in the no PC group; HR, 2.45; 95% CI, 0.81-7.43) and those with refractory disease (5-year survival rate, 41.8% in the PC group vs 40.7% in the no PC group; HR, 0.93; 95% CI, 0.79-1.10).

**Conclusions and Relevance:**

In this cohort study, preoperative chemosensitivity was associated with survival among patients with resectable gastric adenocarcinoma who received PC. These findings may help inform future studies to personalize postoperative therapy.

## Key Points

**Question:**

Is preoperative chemosensitivity associated with increased survival among patients with resectable gastric adenocarcinoma who undergo postoperative chemotherapy?

**Findings:**

In this national cohort study of 2382 patients, postoperative chemotherapy was significantly associated with longer postoperative survival in patients with chemosensitive disease but not in those with very sensitive or refractory disease.

**Meaning:**

These findings suggest that preoperative chemosensitivity may be helpful in making the decision regarding postoperative chemotherapy for patients with resectable gastric adenocarcinoma.

## Teaser

This cohort study examines whether preoperative chemosensitivity is associated with postoperative survival among patients with resectable gastric adenocarcinoma who receive postoperative chemotherapy.

## Introduction

Gastric adenocarcinoma is generally associated with poor prognosis, even at the resectable stage.^1^ Perioperative chemotherapy is a stand of care for locally advanced gastric carcinoma.^1,2^ However, completion of the entire course of postoperative chemotherapy (PC) is challenging. Even in well-selected trial participants, the completion rate is less than 50%.^3^ In addition, the response to preoperative chemotherapy varies. Approximately 10% to 20% of patients are very sensitive to preoperative chemotherapy, leading to complete pathological response.^4,5^ In contrast, at least 20% of patients could achieve only radiologically stable disease.^6^ The current standard postoperative practice remains to complete the same preoperative regimen as tolerated, regardless of the patient’s response to preoperative chemotherapy.^3^

However, the benefit associated with PC is unclear for all patients, considering the low completion rate.^7^ There is currently no biomarker that can reliably select patients who may benefit from PC. In this study using data from the National Cancer Database (NCDB), we hypothesized that preoperative chemosensitivity is associated with survival among patients with resectable gastric adenocarcinoma who also receive PC.

## Methods

### Database and Variables

 Because the data are publicly available and anonymous, the study was exempted from institutional review board review and approval in accordance with 45 CFR §46. Informed consent was also exempted for the same reason. This study follows Strengthening the Reporting of Observational Studies in Epidemiology (STROBE) reporting guidelines.

NCDB is a US national cancer registry that captures more than 70% of newly diagnosed gastric cancers.^8^ Only the first course of treatment is recorded in NCDB, including preoperative and postoperative treatment.^9^

In this cohort study, we included patients from the NCDB who were at least 18 years old at diagnosis, had American Joint Cancer Committee clinical stage II or III disease, received preoperative chemotherapy, underwent curative surgery with negative margins, and did not receive radiotherapy. Patients were excluded if any of the following conditions were met: history of another malignancy, the presence of metastatic disease at the time of pathological staging, or missing pathological staging information. Those who died within 90 days of surgery were also excluded to avoid immortal time bias, because patients need to survive a certain amount of time to start PC.^10^ Ninety days was chosen because it is a metric of surgical care quality.

We extracted from the NCDB demographic characteristics (age, sex, race, and insurance status), Charlson Comorbidity Index score, treating facility type, tumor data (year of diagnosis, grade, histological profile, behavior, American Joint Committee on Cancer clinical and pathological TNM stage, and number of regional nodes examined), treatment information (days from initial diagnosis to definitive surgery, days from initial diagnosis to starting chemotherapy, single or multiple chemotherapy agent, and PC), short-term outcome (days of surgical inpatient stay, 30-day readmission, and 90-day mortality), and survival from initial diagnosis. Overall survival was calculated from surgical discharge until last contact or death. Racial information was collected by participating hospitals of NCDB and was reported in this study because of the known racial disparities in survival among patients with gastric cancer. Treatment pertaining to recurrence and/or progression is not recorded. Invasive gastric adenocarcinoma was identified by *International Classification of Diseases for Oncology, Third Edition* (topographical codes, C160-C169; morphological codes, 8140-8384, 8480, and 8490; behavior code, 3).

The primary outcome in this study is overall survival after surgical discharge. The exposure is PC. Sensitivity to preoperative chemotherapy was defined as follows: very sensitive (ypT0N0), sensitive (pathological less than clinical TNM stage, excluding ypT0N0), and refractory (pathological greater than or equal to clinical TNM stage).

### Statistical Analysis

Data were analyzed in April 2021. Data cleaning was performed with SAS statistical software version 9.4 (SAS Institute) and R statistical software version 4.0.3 (R Project for Statistical Computing). Data analysis was performed with R software. Pearson χ^2^ test was used to compare distribution of categorical data. Kruskal-Wallis rank sum test was used to compare the distribution of numeric data. The gtsummary package in R was used to perform the 2 aforementioned tests and to produce cross-tabulation tables.

The survival and survminer packages in R were used to perform survival analysis and Cox regression. Kaplan-Meier curves were used to estimate survival. A log-rank test was used to compare survival between and among groups. A Cox regression model was used and included the following variables: age, race, sex, insurance status, treating facility type, year of diagnosis, Charlson Comorbidity Index score, tumor grade, primary tumor site, clinical TNM stage, days from initiating preoperative chemotherapy to surgery, sensitivity to preoperative chemotherapy, number of regional nodes examined, 30-day unplanned readmission, and postoperative treatment. Variables that were significant in univariable Cox models of whole group and were significantly associated with receipt of PC in logistic regression were selected for multivariable Cox models. Missing values were omitted in the Cox regression model. Because of the focus of this study, postoperative treatment was always included in the multivariable Cox model. Interaction was tested for the survival association by preoperative chemosensitivity and postoperative treatment. *P* < .05 was considered significant. All hypothesis tests were 2-sided.

## Results

### Patient Characteristics

We identified 2382 patients (1599 men [67%]; median [IQR] age, 63 [54-70] years) between 2006 to 2017. The eFigure in the Supplement shows the flowchart of case selection. Most patients were White (1681 patients [71%]), had no comorbidity (1720 patients [72%]), received no PC (1524 patients [64%]), and received at least a double agent regimen (2171 patients [91%]; 152 patients had no known number of agents). Table 1 shows patient characteristics by preoperative chemosensitivity. Most patients (1483 patients [62%]) had refractory disease, followed by sensitive (727 patients [31%]) and very sensitive (172 patients [7%]) disease. On logistic regression, patients with older age (odds ratio [OR], 0.99; 95% CI, 0.97-1.00), comorbidity (OR, 0.71; 95% CI, 0.57-0.90), longer time from chemotherapy initiation to surgery (OR, 0.99; 95% CI, 0.97-1.00), less sensitivity to preoperative chemotherapy (very sensitive vs refractory OR, 0.58; 95% CI, 0.37-0.89; sensitive vs refractory OR, 0.96; 95% CI, 0.76-1.20), and longer surgical hospitalization (OR, 0.95; 95% CI, 0.93-0.97) had a significantly lower likelihood of receiving PC. (eTable 1 in the Supplement).

**Table 1.** Patient Characteristics by Preoperative Chemosensitivity

Characteristic	Preoperative chemosensitivity, participants, No. (%)			*P* value^a^
	Refractory (n = 1483)	Sensitive (n = 727)	Very sensitive (n = 172)	
Age, y				
<65	844 (57)	388 (53)	94 (55)	.28
≥65	639 (43)	339 (47)	78 (45)	
Sex				
Female	523 (35)	221 (30)	39 (23)	<.001
Male	960 (65)	506 (70)	133 (77)	
Race				
Black	231 (16)	115 (16)	23 (13)	.30
White	1047 (71)	504 (69)	133 (77)	
Other^b^	205 (14)	108 (15)	16 (9.3)	
Diagnosis period				
2006-2011	437 (29)	225 (31)	40 (23)	.14
2012-2017	1046 (71)	502 (69)	132 (77)	
Insurance				
Uninsured	51 (3.5)	25 (3.5)	5 (2.9)	.69
Government	706 (48)	366 (51)	80 (47)	
Private	699 (48)	323 (45)	86 (50)	
Missing	27	13	1	
Treating facility				
Academic or integrated	1055 (75)	516 (74)	117 (69)	.16
Community	343 (25)	183 (26)	53 (31)	
Missing	85	28	2	
Comorbidity				
No comorbidity	1085 (73)	513 (71)	122 (71)	.41
Has comorbidity	398 (27)	214 (29)	50 (29)	
Grade				
I-II	325 (24)	236 (35)	44 (32)	<.001
III-IV	1045 (76)	433 (65)	95 (68)	
Missing	113	58	33	
Clinical TNM stage				
II	1025 (69)	363 (50)	97 (56)	<.001
III	458 (31)	364 (50)	75 (44)	
Time from chemotherapy initiation to surgery, median (IQR), d	98 (84-115)	99 (86-116)	98 (88-111)	.17
Missing	108	40	6	
Nodes examined, No.				
<15	338 (23)	210 (29)	53 (31)	.002
≥15	1128 (77)	509 (71)	116 (69)	
Missing	17	8	3	
Unplanned 30-d readmission	73 (4.9)	33 (4.5)	6 (3.5)	.68
Duration of surgical hospitalization, median (IQR), d	8 (6-11)	7 (6-10)	7 (6-9)	.004
Missing	133	54	15	
Postoperative treatment				
No chemotherapy	924 (62)	472 (65)	128 (74)	.006
Chemotherapy	559 (38)	255 (35)	44 (26)	

^a^ *P* values were calculated with Pearson χ^2^ test or Kruskal-Wallis rank sum test.

^b^ Other refers to American Indian, Asian, Pacific Islander, multiracial, and any other race.

### Survival by Preoperative Chemosensitivity and PC Use

After a median follow-up of 34 months, patients with refractory disease had the worst survival compared with those with sensitive disease (Cox hazard ratio [HR], 0.39; 95% CI, 0.32-0.46) and very sensitive disease (Cox HR, 0.12; 95% CI, 0.07-0.20; log-rank *P* < .001) (Figure, panel A, and eTable 2 in the Supplement). Receipt of PC was not associated with improved survival in the whole group (Cox HR, 0.88; 95% CI, 0.75-1.02; log-rank *P* = .37) (Figure, panel B, and eTable 2 in the Supplement).

**Figure.**
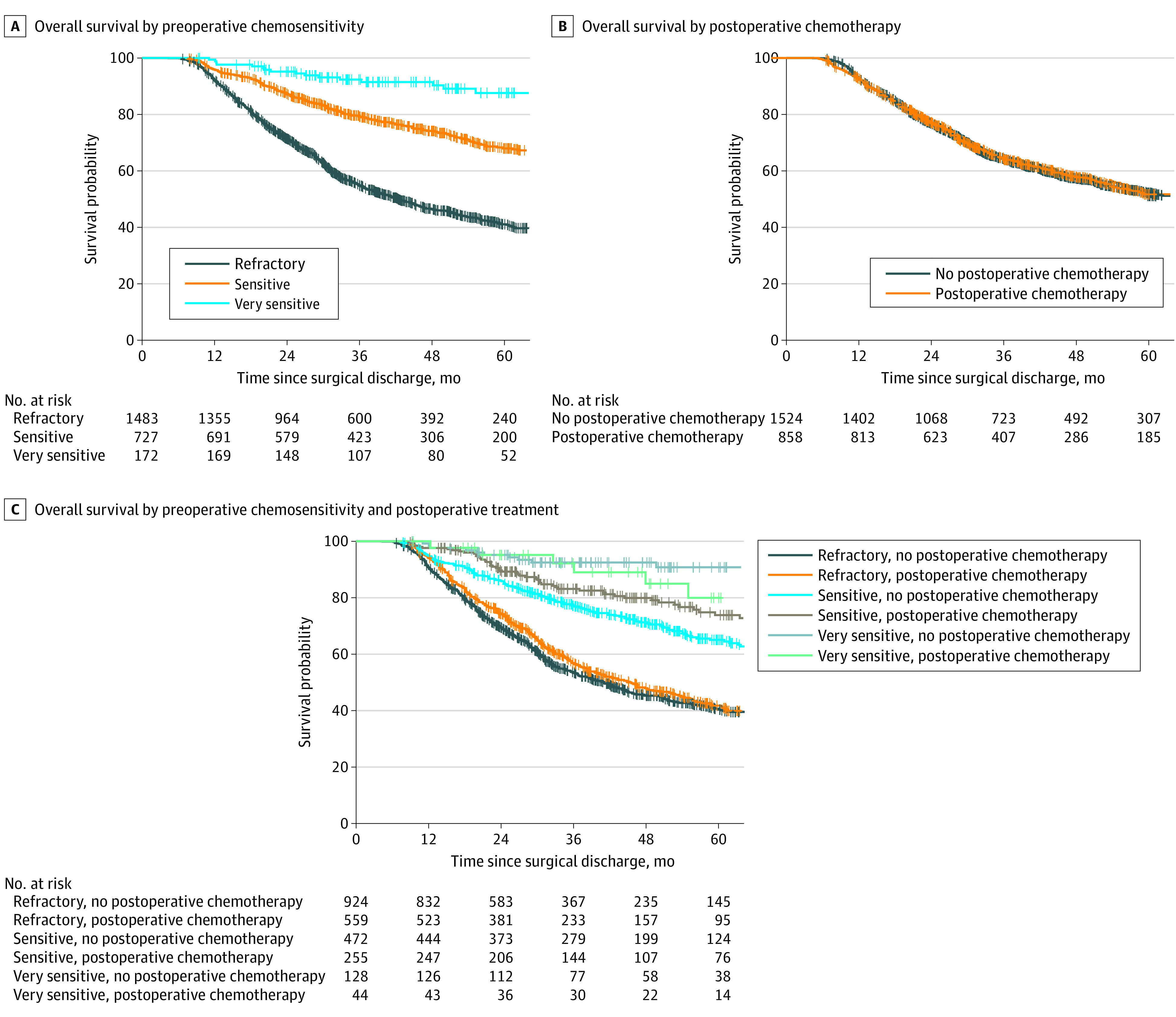
Kaplan-Meier Curves for Overall Survival in Patients With Resected Clinical Stage II to III Gastric Adenocarcinoma Who Received Preoperative Chemotherapy and Surgery Graphs show patients stratified by preoperative chemosensitivity (A), postoperative treatment (B), and preoperative chemosensitivity and postoperative treatment (C).

### Survival by Postoperative Treatment Stratified by Preoperative Chemosensitivity

eTable 3 in the Supplement shows patient characteristics by preoperative chemosensitivity and PC. There was significant interaction between the 2 factors for survival (*P *for interaction = .03) (eTable 4 in the Supplement; Figure, panel C).

PC was associated with longer overall survival in patients with sensitive disease (Cox HR, 0.64; 95% CI, 0.46-0.91; log-rank *P* = .02) (Figure, panel C, and Table 2). In the PC group, the survival rates were 97.6% at 1 year, 83.1% at 3 years, and 73.8% at 5 years. In the no PC group, survival rates were 94.9% at 1 year, 77.2% at 3 years, and 65.0% at 5 years. In contrast, overall survival was not significantly associated with PC among patients with very sensitive disease (5-year survival rate, 80.0% in the PC group vs 90.8% in the no PC group; HR, 2.45; 95% CI, 0.81-7.43) or those with refractory disease (5-year survival rate, 41.8% in the PC group vs 40.7% in the no PC group; HR, 0.93; 95% CI, 0.79-1.10) (Figure, panel C, and Table 2).

**Table 2.** HR of Postoperative Treatment by Preoperative Chemosensitivity^a^

Preoperative chemosensitivity	Postoperative chemotherapy vs none, HR (95% CI)	*P* value
Refractory	0.93 (0.79-1.10)	.41
Sensitive	0.64 (0.46-0.91)	.01
Very sensitive	2.45 (0.81-7.43)	.11

Abbreviation: HR, hazard ratio.

^a^ Full Cox model with main and interaction effects is listed in eTable 4 in the Supplement.

## Discussion

This cohort study found that preoperative chemosensitivity was associated with survival among patients with resectable gastric adenocarcinoma who received PC. Preoperative chemosensitivity was also associated with postoperative survival. PC was not found to be associated with improved survival in the whole group. However, the benefit associated with PC differed among patients with different preoperative chemosensitivity. Patients with sensitive disease, defined as pathological less than clinical stage (excluding complete pathological response), had improved survival associated with receipt of PC compared with no PC. Patients with very sensitive and refractory disease were not found to have improved survival associated with PC.

These findings are valuable for personalizing postoperative treatment in patients with resectable gastric adenocarcinoma, considering the low completion rate of the entire PC course in those patients associated with postoperative recovery and toxic effects of PC. A trial by Al-Batran et al^3^ showed that no more than 60% of patients were able to receive PC, and no more than one-half of all patients could complete all cycles of chemotherapy. An NCDB study^11^ showed that among patients who did not receive any neoadjuvant treatment, only 29% received PC, among whom only 58% were able to receive PC within 8 weeks of surgery.

Our results can be explained by the biological rationale of PC, which eradicates possible micrometastases after definitive surgery. In this regard, patients who would benefit from PC are those who still have micrometastases after surgery and whose cancers are sensitive to the chemotherapy regimen. In current practice, the PC regimen is typically the same as the preoperative one regardless of preoperative chemosensitivity. It is plausible that micrometastases, if any, in patients with sensitive disease remain responsive to the same postoperative regimen. Administration of PC would more likely provide benefit in such a situation. On the other hand, it is less likely that micrometastases would respond to PC if the primary tumor is resistant to the same preoperative treatment as in patients with refractory disease in our study. Furthermore, patients with very sensitive disease, who have achieved complete pathological response at the primary site, may have micrometastases already eradicated by preoperative chemotherapy alone, considering the exceptional response at the primary site.

Efforts have also been devoted to identifying subgroups who may benefit from PC, with conflicting results.^12,13^ Nodal metastasis after chemotherapy is associated with increased risk of poor prognosis, but our study did not show any interaction between yp nodal status and postoperative survival benefit (eTable 5 in the Supplement). Novel approaches, including circulating tumor DNA (ctDNA), have been explored in resected gastric cancer.^14^ ctDNA from patients in the CRITICS trial^14^ was shown to be associated with event-free survival and pathological response. Along with ctDNA, genomic signatures and radiomics are other advanced technologies of great potential, but they are costly, complex, and have not been rigorously assessed. In contrast, our tool to assess chemosensitivity is simple, intuitive, and requires no additional cost. It can be quickly adopted clinically if it is prospectively validated. Extrapolating from our results, dynamic change of ctDNA burden may also be incorporated into preoperative chemosensitivity assessment in future studies. Our findings have important implications in the design of future research to personalize PC.

### Limitations

One of the known inherent NCDB limitations is that chemotherapy information may be missing if it was administered outside the reporting facility, so that some patients in the PC group may be incorrectly classified as being in the no PC group. However, because our patients have received preoperative chemotherapy, the likelihood of receiving PC in another facility would be much lower. NCDB only recorded the first administration date of chemotherapy, so the exact time point of PC is unknown in patients who have received preoperative chemotherapy. The staging modality is also unknown, and clinical staging can be inaccurate. Although 91% of patients received at least 2 agents, the exact regimen and number of cycles are unknown; however, the impact of regimen is likely to be similar across all chemosensitivity groups. The nature of the retrospective study also predisposes results to other unknown confounding factors for which we cannot adjust.

## Conclusions

In conclusion, this study found that preoperative chemosensitivity is associated with survival among patients with clinical stage II to III gastric adenocarcinoma who receive PC. A large number of patients and real-world data make our results generalizable. Preoperative chemosensitivity may be incorporated into the decision-making process of PC administration. Our findings have the potential to inform future prospective studies to personalize postoperative therapy.

## Notes

Supplement. **eFigure.** Flowchart of Study **eTable 1.** Logistic Regression Model for Receiving Postoperative Chemotherapy **eTable 2.** Univariable and Multivariable Cox Regression Models for Survival Without Interaction Effects **eTable 3.** Patient Characteristics by Preoperative Chemosensitivity and Postoperative Chemotherapy **eTable 4.** Cox Model of Overall Survival with Interaction Effect of Preoperative Chemosensitivity and Postoperative Chemotherapy **eTable 5.** Association of Survival in Patients Treated With Postoperative Chemotherapy or Not by yp Nodal Status
