# Supplementary material for: Association of Preoperative Chemosensitivity With Postoperative Survival in Patients With Resected Gastric Adenocarcinoma
Source: JAMA Netw Open. 2021 Nov 19;4(11):e2135340. doi: 10.1001/jamanetworkopen.2021.35340 (PMC8605482; doi:10.1001/jamanetworkopen.2021.35340)
Supplement: Supplement. — eFigure. Flowchart of Study eTable 1. Logistic Regression Model for Receiving Postoperative Chemotherapy eTable 2. Univariable and Multivariable Cox Regression Models for Survival Without Interaction Effects eTable 3. Patient Characteristics by Preoperative Chemosensitivity and Postoperative Chemotherapy eTable 4. Cox Model of Overall Survival with Interaction Effect of Preoperative Chemosensitivity and Postoperative Chemotherapy eTable 5. Association of Survival in Patients Treated With Postoperative Chemotherapy or Not by yp Nodal Status [file jamanetwopen-e2135340-s001.pdf]

Supplemental Online Content

Deng L, Groman A, Jiang C, et al. Association of preoperative chemosensitivity with postoperative survival in patients with resected gastric adenocarcinoma. *JAMA Netw Open*. 2021;4(11):e2135340. doi:10.1001/jamanetworkopen.2021.35340

eFigure. Flowchart of Study

eTable 1. Logistic Regression Model for Receiving Postoperative Chemotherapy

eTable 2. Univariable and Multivariable Cox Regression Models for Survival Without Interaction Effects

eTable 3. Patient Characteristics by Preoperative Chemosensitivity and Postoperative Chemotherapy

eTable 4. Cox Model of Overall Survival with Interaction Effect of Preoperative Chemosensitivity and Postoperative Chemotherapy

eTable 5. Association of Survival in Patients Treated With Postoperative Chemotherapy or Not by yp Nodal Status

This supplemental material has been provided by the authors to give readers additional information about their work.

© 2021 Deng L et al. *JAMA Network Open.*

eFigure. Flowchart of Study

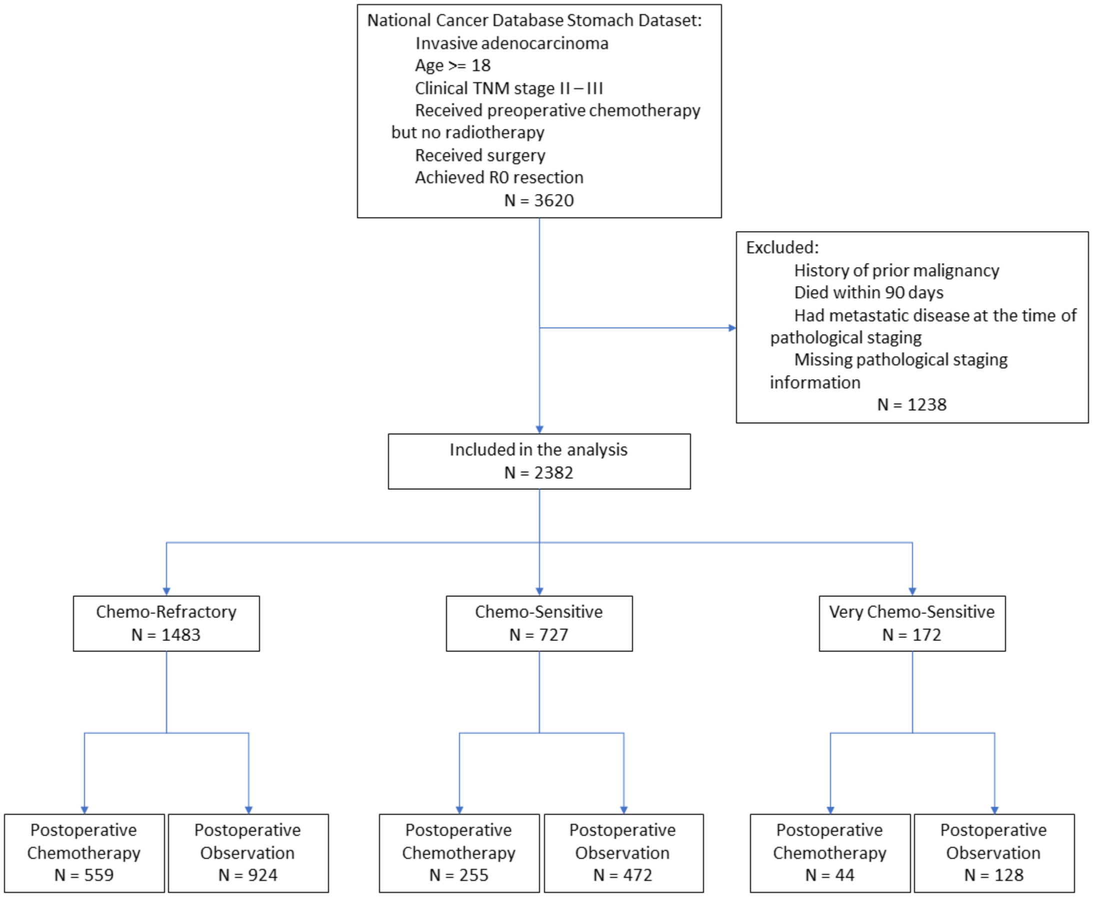

© 2021 Deng L et al. *JAMA Network Open.*

eTable 1. Logistic Regression Model for Receiving Postoperative Chemotherapy

**Variables**	**OR**[1]	**95% CI**[1]	**p-value**
**Age (continuous)**	0.99	0.97 - 1.00	**0.018**
**Race**			0.721
White	—	—	
Black	0.91	0.68 - 1.21	
Other	1.05	0.78 - 1.43	
**Sex**			0.303
Female	—	—	
Male	1.12	0.90 - 1.40	
**Insurance**			0.724
Uninsured	—	—	
Government	0.91	0.52 - 1.61	
Private	1.00	0.58 - 1.75	
**Treating Facility**			0.16
Academic/Integrated	—	—	
Community	1.19	0.93 - 1.52	
**Year of Diagnosis (continuous)**	1.03	0.99 - 1.08	0.175
**Comorbidity**			**0.004**
No Comorbidity	—	—	
Has Comorbidity	0.71	0.57 - 0.90	
**Grade**			0.103
I/II	—	—	
III/IV	1.21	0.96 - 1.53	
**Clinical TNM Stage**			0.172
II	—	—	
III	1.16	0.94 - 1.45	
**Days from Chemotherapy Initiation to Surgery (continuous)**	0.99	0.99 - 1.00	**<0.001**
**# of Nodes Examined**			0.153
<15	—	—	
15	1.20	0.94 - 1.53	
**Preoperative Chemosensitivity**			**0.041**
Refractory	—	—	

© 2021 Deng L et al. *JAMA Network Open.*

Sensitive	0.96	0.76 - 1.20	
Very Sensitive	0.58	0.37 - 0.89	
**Days of Surgical Hospitalization (continuous)**	0.95	0.93 - 0.97	**<0.001**
**Unplanned 30-day Readmission**			0.58
No	—	—	
Yes	0.87	0.53 - 1.40	
[1] OR = Odds Ratio, CI = Confidence Interval			

© 2021 Deng L et al. *JAMA Network Open.*

eTable 2. Univariable and Multivariable Cox Regression Models for Survival Without Interaction Effects

**Variables**	**Univariable**		**Multivariable**	
	Hazard Ratio	p value	Hazard Ratio	p value
**Age (continuous)**	1.00	0.201	1.00	0.158
**Race**				
White	Reference			
Black	0.69	**<0.001**	0.84	**0.102**
Other	0.51	**<0.001**	0.58	**<0.001**
**Male vs. Female**	1.06	0.370	—	—
**Insurance**			—	—
Uninsured	Reference			
Government	1.00	0.987		
Private	0.91	0.596		
**Community vs. Academic/Integrated Program**	1.07	0.340	—	—
**Year of diagnosis (continuous)**	0.93	**<0.001**	0.94	**<0.001**
**Has vs. No Comorbidity**	1.05	0.510	1.10	0.236
**Tumor Grade III/IV vs. II/I**	1.58	**<0.001**	1.59	**<0.001**
**Clinical TNM stage III vs. II**	1.35	**<0.001**	1.63	**<0.001**
**Days from initiating preoperative Chemotherapy to Surgery (continuous)**	1.00	0.370	1.00	0.708
**≥ 15 vs. < 15 Regional Lymph Nodes Examined**	0.83	**0.006**	0.76	**<0.001**
**Preoperative Chemosensitivity**				
Refractory	Reference			
Sensitive	0.42	**<0.001**	0.39	**<0.001**
Very Sensitive	0.17	**<0.001**	0.12	**<0.001**
**Days of Surgical Hospitalization (continuous)**	1.01	**<0.001**	1.01	**<0.001**
**Unplanned vs. No Unplanned 30-day Readmission**	1.11	0.490	—	—
**Postoperative Chemotherapy vs. None**	0.94	0.370	0.88	0.091

© 2021 Deng L et al. *JAMA Network Open.*

eTable 3. Patient Characteristics by Preoperative Chemosensitivity and Postoperative Chemotherapy

**Characteristic**	**Very Sensitive**			**Sensitive**			**Refractory**		
	**No Chemotherapy**, N = 128[1]	**Chemotherapy**, N = 44[1]	**p-value**[2]	**No Chemotherapy**, N = 472[1]	**Chemotherapy**, N = 255[1]	**p-value**[2]	**No Chemotherapy**, N = 924[1]	**Chemotherapy**, N = 559[1]	**p-value**[2]
**Age (Year)**			**0.015**			**<0.001**			**0.001**
<65	63 (49%)	31 (70%)		229 (49%)	159 (62%)		496 (54%)	348 (62%)	
65	65 (51%)	13 (30%)		243 (51%)	96 (38%)		428 (46%)	211 (38%)	
**Sex**			0.684			0.798			0.665
Female	30 (23%)	9 (20%)		145 (31%)	76 (30%)		322 (35%)	201 (36%)	
Male	98 (77%)	35 (80%)		327 (69%)	179 (70%)		602 (65%)	358 (64%)	
**Race**			0.534			0.392			**0.003**
White	100 (78%)	33 (75%)		321 (68%)	183 (72%)		665 (72%)	382 (68%)	
Black	15 (12%)	8 (18%)		81 (17%)	34 (13%)		153 (17%)	78 (14%)	
Other	13 (10%)	3 (6.8%)		70 (15%)	38 (15%)		106 (11%)	99 (18%)	
**Diagnosis Period**			0.465			0.408			0.305
2006-2011	28 (22%)	12 (27%)		151 (32%)	74 (29%)		281 (30%)	156 (28%)	
2012-2017	100 (78%)	32 (73%)		321 (68%)	181 (71%)		643 (70%)	403 (72%)	
**Insurance**			0.051			0.161			**<0.001**
Uninsured	3 (2.4%)	2 (4.5%)		15 (3.2%)	10 (4.0%)		29 (3.2%)	22 (4.0%)	
Government	66 (52%)	14 (32%)		249 (54%)	117 (46%)		483 (53%)	223 (41%)	
Private	58 (46%)	28 (64%)		198 (43%)	125 (50%)		394 (43%)	305 (55%)	
(Missing)	1	0		10	3		18	9	

© 2021 Deng L et al. *JAMA Network Open.*

**Treating Facility**			0.821			0.702			0.279
Academic/Integrated	88 (69%)	29 (67%)		338 (74%)	178 (73%)		671 (76%)	384 (74%)	
Community	39 (31%)	14 (33%)		117 (26%)	66 (27%)		207 (24%)	136 (26%)	
(Missing)	1	1		17	11		46	39	
**Comorbidity**			**0.026**			0.122			**0.004**
No Comorbidity	85 (66%)	37 (84%)		324 (69%)	189 (74%)		652 (71%)	433 (77%)	
Has Comorbidity	43 (34%)	7 (16%)		148 (31%)	66 (26%)		272 (29%)	126 (23%)	
**Grade**			0.585			0.131			0.175
I/II	33 (33%)	11 (28%)		162 (37%)	74 (31%)		212 (25%)	113 (22%)	
III/IV	67 (67%)	28 (72%)		272 (63%)	161 (69%)		638 (75%)	407 (78%)	
(Missing)	28	5		38	20		74	39	
**Clinical TNM Stage**			0.774			0.326			0.697
II	73 (57%)	24 (55%)		242 (51%)	121 (47%)		642 (69%)	383 (69%)	
III	55 (43%)	20 (45%)		230 (49%)	134 (53%)		282 (31%)	176 (31%)	
**Days from Chemotherapy Initiation to Surgery**	98 (89, 114)	97 (85, 102)	0.086	101 (88, 123)	97 (85, 108)	**<0.001**	100 (85, 119)	95 (82, 111)	**<0.001**
(Missing)	6	0		32	8		88	20	
**# of Nodes Examined**			0.344			0.052			0.303
<15	42 (33%)	11 (26%)		148 (32%)	62 (25%)		219 (24%)	119 (22%)	
15	84 (67%)	32 (74%)		320 (68%)	189 (75%)		696 (76%)	432 (78%)	

© 2021 Deng L et al. *JAMA Network Open.*

(Missing)	2	1		4	4		9	8	
**Unplanned 30-day Readmission**	6 (4.7%)	0 (0%)	0.34	22 (4.7%)	11 (4.3%)	0.83	46 (5.0%)	27 (4.8%)	0.898
**Days of Surgical Hospitalization**	7.0 (5.0, 9.0)	7.0 (6.0, 9.2)	0.667	8.0 (6.0, 11.0)	7.0 (5.0, 9.0)	**0.003**	8 (6, 12)	7 (6, 10)	**<0.001**
(Missing)	11	4		43	11		80	53	

[1] n (%); Median (IQR)

[2] Pearson's Chi-squared test; Fisher's exact test; Wilcoxon rank sum test

© 2021 Deng L et al. *JAMA Network Open.*

eTable 4. Cox Model of Overall Survival with Interaction Effect of Preoperative Chemosensitivity and Postoperative Chemotherapy

Characteristic	HR	95% CI	p-value
**Age (continuous)**	1.00	1.00 - 1.01	0.14
**Year of Diagnosis (continuous)**	0.94	0.91 - 0.97	<0.001
**Comorbidity**			0.21
No Comorbidity	—	—	
Has Comorbidity	1.11	0.94 - 1.30	
**Race**			<0.001
White	—	—	
Black	0.83	0.67 - 1.03	
Other	0.58	0.44 - 0.75	
**Tumor Grade**			<0.001
I/II	—	—	
III/IV	1.59	1.34 - 1.90	
**Clinical TNM Stage**			<0.001
II	—	—	
III	1.63	1.41 - 1.90	

© 2021 Deng L et al. *JAMA Network Open.*

**Days from Chemotherapy Initiation to Surgery (continuous)**	1.00	1.00 - 1.00	0.65
**Days of Surgical Hospitalization (continuous)**	1.02	1.01 - 1.02	<0.001
**# of Nodes Examined**			0.001
<15	—	—	
=15	0.76	0.65 - 0.89	
**Preoperative Chemosensitivity**			<0.001
Refractory	—	—	
Sensitive	0.44	0.35 - 0.54	
Very Sensitive	0.08	0.04 - 0.18	
**Postoperative Chemotherapy**			0.41
No Chemotherapy	—	—	
Chemotherapy	0.93	0.79 - 1.10	
**Preoperative Chemosensitivity * Postoperative Chemotherapy (interaction effect)**			0.033
Sensitive * Chemotherapy	0.69	0.47 - 1.01	
Very Sensitive * Chemotherapy	2.63	0.86 - 8.06	

HR = Hazard Ratio, CI = Confidence Interval

© 2021 Deng L et al. *JAMA Network Open.*

eTable 5. Association of Survival in Patients Treated With Postoperative Chemotherapy or Not by yp Nodal Status

	Number of patients	5 – year survival rate (Chemotherapy vs. No)	Log-rank p value	Cox p value
ypCR	172	80.0% vs. 90.8%	0.223	0.143
ypN0	993	72.0% vs. 65.7%	0.051	0.213
ypN+	1241	41.5% vs. 38.1%	0.158	0.078

© 2021 Deng L et al. *JAMA Network Open.*
